# Physical activity and nutrition in relation to resilience: a cross-sectional study

**DOI:** 10.1038/s41598-024-52753-6

**Published:** 2024-01-27

**Authors:** Bernhard Leipold, Kristina Klier, Ellen Dapperger, Annette Schmidt

**Affiliations:** 1 Institute of Psychology, University of the Bundeswehr Munich, Werner-Heisenberg-Weg 39, 85577 Neubiberg, Germany; 2 Institute of Sport Science, University of the Bundeswehr Munich, Werner-Heisenberg-Weg 39, 85577 Neubiberg, Germany

**Keywords:** Psychology, Health care, Risk factors

## Abstract

A healthy lifestyle is often discussed as being a characteristic of or a prerequisite for quality of life. In phases of high subjective stress (work overload, negative thoughts), however, its protective function can be limited. The two present survey studies examined two facets of a health-related lifestyle (physical activity and nutritional awareness), in particular, the correlations with general life satisfaction and their adaptive function in respect to stress (resilience). In addition, because episodes of increased stress can have a negative effect on eating, the interactions with the consumption of less healthy food were examined. Two cross-sectional studies were conducted successively with adults aged between 18 and 72 in Germany. *Study 1* (*N* = 685) examined the research questions with correlations, moderated regression analyses, and structural equation models. *Study 2* (*N* = 628) differentiated between sport, occupational and daily activities. *Study 1* showed that the amount of physical activity and nutritional awareness are correlated with life satisfaction. The relationship between stress appraisals and general life satisfaction was moderated by physical activity and nutritional awareness (stress-buffer effect). *Study 2* replicated the stress-buffer effects of nutritional awareness, daily activities, and occupational activities. Both studies showed that stress is associated with consumption of less healthy food and found interactions with physical activity and nutritional awareness. Discussed are the adaptive role of physical activity and nutritional awareness in times of stress.

**Trial Registration** EK UniBw M 23-06, 12/16/2022.

## Introduction

It is in the nature of humans to aim for high subjective well-being and optimal health^1^. According to Diener’s definition, the concept of subjective well-being includes fulfilling life while feeling good^2^ and was often synonymously used for happiness or life satisfaction^3^. However, research advancing a more differentiated view denotes life satisfaction as the cognitive component of subjective well-being and happiness as the affective component^4^. From a salutogenetic perspective, health is not only understood as being free from disease but also as homeostasis of stressors and resources^5^. This makes the concept of resilience become relevant—the ability of individuals and systems to successfully adapt to threats and losses^6–8^.

In the following we refer to a relational concept of resilience^9^ that differentiates between adversity (perceived stress), resources (e.g., adaptive processes, coping strategies, healthy lifestyle), and outcomes (e.g., life satisfaction). Many studies have examined the relationship between well-being and physical activity (e.g.,^10,11^) or health-related nutrition (e.g.,^12,13^). We examine resilience as a correlational relationship within a variable-focused approach^14^ in which risk factors, moderators, and outcome variables are distinguished. The interaction between a risk factor and a moderator will be used as a marker of resilience. In cross-sectional studies, one can only speculate as to the direction of effects and the actual tackling of stressors. This should be considered when interpreting the findings. On the basis of the transtheoretical stress model according to which stress appraisals and coping resources predict outcome variables^15,16^, the present study examines the protective function of a health-related lifestyle (moderator) in relation to subjective stress (risk factor) and general life satisfaction (outcome variable). In detail, we examine whether physical activity (sport, occupational or daily activities^17^) or nutritional awareness (whether people watch what they eat or follow a nutritional concept) moderate the negative association between subjective stress and general life satisfaction (as a sample case of resilience). Very little research has focused on the stress-buffering role of nutrition and physical activity. In addition, interactions with the consumption of less healthy food are examined.

## Physical activity, nutrition, and well-being

Several studies have shown a direct link between lifestyle (i.e. health behaviors) and life satisfaction or subjective well-being^11,18,19^. For example, Maher and colleagues^18^ have examined how life satisfaction varies over the lifespan. In their diary study with 150 participants (18–89 years old) over about one year, they found physical activity to be a predictor of physical and mental health. Although daily changes in physical activity and life satisfaction occur, they have shown a significant positive association between regular physical activity and life satisfaction. Similar results have been found by An et al.^4^, in whose study the level of physical activity significantly correlated with higher life satisfaction and happiness in young, middle, as well as older adults. Panza et al.^20^ have correlated sedentary behavior with reduced well-being. Even though the effects of the intensity of activity differ between person, individuals meeting the activity guidelines of the World Health Organization generally report higher well-being^21,22^. On the other hand, several studies demonstrated no evidence for a strong association between physical activity and mental well-being^10,18,23^.

A mixed pattern of results can also be found in research on the association between nutrition and well-being. For instance, in the Norwegian Health Study (*N* = 1619, 65 + years old), which examined the interaction of a healthy lifestyle, more precisely food patterns, and life satisfaction, healthy food-pattern participants reported significantly higher quality of life and lower anxiety and depression^12^. In line with this, Tan et al.^24^ have found a small but significant correlation between healthy nutrition and higher well-being. Especially in the elderly, eating behavior seems to correlate with quality of life as well as survival. These findings are also in line with a study by Yau and Potenza^13^, according to whom unhealthy behavior reduces life satisfaction and can cause illnesses. This is of particular importance when looking at the interaction between stress, eating behavior, neurobiological adaptions and obesity. Besides sufficient fruit/vegetable intake, sociodemographic factors as well as the presence of (chronic) disease appear to be highly influencing mediators of life satisfaction^19^.

Although there are studies that have shown the link between activity, nutritional awareness and life satisfaction, some studies have provided little or no evidence of this (e.g.,^18,23,25^). Thus, in addition to bivariate associations, we want to investigate the stress-buffering effects of physical activity and nutritional awareness, which have been studied less frequently.

## A healthy lifestyle in times of stress: a sample case of resilience

Resilience is defined as the potential to recover from adverse circumstances. We concentrate on a perspective that emphasizes the relational structure of resilience^9,26^ and differentiates between adversity (perceived stressors), adaptive processes (e.g., coping, healthy lifestyle), and criteria (e.g., life satisfaction, well-being, self-esteem). Concepts of a healthy lifestyle^24^ or self-regulation^27^ emphasize the active role of the self, and resilience refers to the resulting stabilization of the individual’s well-being against stress and losses^6,7,28^. On the basis of these models, we used resilience as an avenue to examine the moderating (stress-buffering) effects of physical activity and nutritional awareness on the relationship between perceived stress and general life satisfaction.

A few studies have found a significant stress buffering effect of physical activity on the relationship between stress and life satisfaction^29^ and health^30^, but the empirical evidence is not consistent. Some studies provided partial support for a stress-buffering effect of physical activity and well-being^31^, for negative but not for positive affect^32^, or found a stress-buffering effect only when intrinsic motivation was high^23^.

Overall, there are many studies that show a buffering effect of physical activity, however, the predictors and outcomes vary and there are still conflicting findings (for an overview see^33^). Many studies focus on psychobiological markers (e.g.,^34,35^) and mental health variables (e.g., depression and burnout;^36,37^), but seldom life satisfaction.

In addition, we wanted to examine the moderating role of nutritional awareness. Nutritional awareness has been found to be important in understanding the impact of stress on well-being^38^. Because activity and nutritional awareness may be utilized differently by different people^35,39^, it would make sense to test whether they moderate the relationship between stress and well-being. To our knowledge, no study has investigated the stress buffering effect of nutritional awareness on life satisfaction.

## Less healthy food consumption in times of stress

Times of increased stress are not only suitable for demonstrating resilience phenomena, but also involve the risk of people resorting to unhealthy diets^40^. In the present study, we make a distinction between nutritional awareness and the consumption of less healthy food (fast food, sweets, higher meat consumption). Mainly in phases of high subjective stress (e.g., work overload, negative thoughts), persons turn to high fat or high sugar food to compensate or reduce stress^13,41^. Ans and colleagues^42^ have shown stress-related changed eating behavior as risk factor for the development of obesity due to changed neurohormonal regulation of appetite. Based on study results that have shown an association between negative emotions or stress and poor nutrition (see^43–45^), we expect that the relationship between eating habits and nutritional awareness is moderated by the perceived level of stress. We expect that the negative correlation between nutritional awareness and less healthy nutrition habits is weakened in times of stress.

## The present study

Although various studies have examined either the relationship of physical activity or nutrition with well-being and health, the interaction of these aspects is still unclear. Thus, the present study examines physical activity and nutritional awareness in relation to resilience and well-being in adulthood. Based on the background described, we assume (1) that physical activity and nutritional awareness are correlated with life satisfaction. We assume (2) a protective effect of physical activity and nutritional awareness on well-being, that is, that both moderate the negative relationship between current subjective stress and general life satisfaction. In addition, we expect (3) that subjective stress is correlated with the consumption of less healthy food/junk food. Finally, we assume that (4) the negative association between nutritional awareness and the consumption of less healthy food is weaker or absent in times of a high degree of subjective stress, because it is then more difficult to stick to one’s resolutions. We started with a cross-sectional study in 2022 in which physical activity was assessed with a global measure. Because it became important for the interpretation of the results to distinguish between the situations in which people were physically active, we conducted *Study 2*, in which we specifically asked the level of activity in each of the three areas (sport, occupational or daily activities).

## Study 1

### Methods

#### Participants

The data was collected during the first half of 2022. Participants between the ages of 18 up to about 70 years were recruited from an online-platform for social research and via advertisement in student classes in Germany. The students received course credit for participation, other participants received approximately € 4. Initially, 749 participants responded to the survey, but 54 could not be considered due to questionable validity of the responses (unreasonably fast completion, incomplete or monotonous responding). Ten participants had to be excluded because they were physically disabled or their responses were outliers with extreme z scores ( >|+ − 3|). The final sample consisted of 685 participants aged 24–72 years (*M* = 48.95, *SD* = 11.25); 52% of the sample were female; males and females did not differ significantly in age, t(681) = 1.23, *p* = 0.22. The body mass index was *M* = 26.58 (*SD* = 5.51). Seventy-four percent practise at least one sport regularly. Jogging (*N* = 170) and cycling (*N* = 144) were the most frequently mentioned activities. Data were checked to ensure the assumptions of normality and outliers. Skew and kurtosis values were below ± 1 for the main variables (see Table 1).

**Table 1:** Descriptive statistics and bivariate correlations.

Variable	M (SD)	Range	α	Skewness/kurtosis	(2)	(3)	(4)	(5)	(6)	(7)
(1) Age	48.94 (11.25)	24–72		− 0.11/− 0.68	− 0.05	− 0.09*	− 0.31***	− 0.19***	− 0.35***	− 0.03
(2) Gender 1 = m, 2 = f				− 0.03/− 1.83	1	0.11**	− 0.14***	− 0.04	0.09*	0.02
(3) Nutritional awareness	0.85 (0.51)	0–2	0.64	− 0.12/− 0.79		1	− 0.19***	0.29***	0.03	0.16***
(4) Less healthy food	3.65 (1.10)	1–7	0.75	0.17/− 0.15			1	0.17***	0.38***	− 0.05
(5) Amount of physical activity	3.61 (1.04)	1–7	0.74	− 0.16/0.06				1	0.06	0.38***
(6) Subjective stress appraisal	3.59 (1.35)	1–7	0.89	0.04/− 0.63					1	− 0.31***
(7) Life satisfaction	4.77 (1.34)	1–7	0.91	− 0.70/− 0.11						1

*N* = 685; *p < 0.05; **p < 0.01; ***p < .001.

#### Measurements

##### Physical activity

Nine items adapted from an instrument developed by Fuchs, Klaperski, Gerber, and Seelig^17^ were used. Fuchs et al.^17^ differentiated between sport, occupational or daily activities. We were interested in a more global value and asked participants to think about the frequency of their activities over the past four weeks (*1* = very seldom to *7* = very often). They were asked to think about and include all their activitites over the past four weeks, whether at home or at work, as well as sport and leisure activites. Items were physically demanding (house)work, physically demanding caregiving tasks, gardening, intensive exercise in everyday life, moderate execise in everyday life, athletic activities, cycling, walking (Cronbach’s alpha was 0.75).

##### Less healthy foods/junk food

Due to time constraints, we focused on single items to measure the types of less healthy foods (e.g., fast food, sweets, higher meat consumption) instead of using rather long nutrition inventories. In detail, participants were asked how often they had eaten the following foods in the past four weeks (*1* = very seldom to *7* = very often): (1) Fast food (e.g., burger, french fries), (2) Junk food/sweets (e.g., cake, biscuits, soft drinks, iced tea), (3) Junk food/snacks (e.g., crisps, pretzels), (4) Meat. In addition, (5) Adding sugar to foods/drinks was assessed. Scores of these five indicators were entered into a factor analysis. Using principal components, one factor was extracted, accounting for 47% of the variance. The mean value was computed (α = 0.71).

##### Nutritional awareness

Four items were used to measure the degree to which participants pay attention to their diet. “Do you watch what you eat?” (*0* = no, *1* = sometimes, *2* = yes), “Please provide the reasons (e.g., body cult, health, improving physical fitness, maintaining physical fitness)”, the number of reasons was used (0, 1, 2 or more reasons), “Do you follow a dietary concept (e.g., healthy diet, healthy eating, attention to nutritional values)?” (*0* = no, *1* = sometimes, *2* = yes), “Do you eat according to a specific nutritional concept (e.g., vegan, low carb, Paleo)?”, the number (0, 1, 2 or more) was used. Scores of these four indicators were entered into a factor analysis. Using principal components, one factor was extracted, accounting for 48% of the variance. We computed the mean value (α = 0.64).

##### Subjective stress

Subjective stress was measured with a shortened and adapted version of the Trier stress inventory^46^. The original scale consists of 39 items assessing facets of chronic stress: Worries, intrusive memories, work-related stress (work overload, work discontent), and social stress (social conflicts, lack of social recognition). To make the time window comparable to that of acivity and nutrition, participants were asked to rate how often they have experienced stress episodes during the last four weeks on a scale from *1* (very seldom) to *7* (very often). Example items are “I had intrusive thoughts about an unpleasant experience” or “I had no time for recreation”. We used 18 items from four facets. Because the four subscales were highly intercorrelated (*r*´s between 0.61 and 0.84), a global stress score was computed (α = 0.93).

##### Life satisfaction

Life satisfaction was assessed with the *Satisfaction With Life Scale* (SWLS;^47^). Participants were asked to rate how well each item applied to them in general (e.g., “I am satisfied with my life”) on a seven-point Likert scale; α = 0.91.

All calculations were conducted using SPSS version 27 and LISREL9.3. We computed bivariate correlations between the main variables. Because age and gender were significantly associated with some of the central constructs, they were used as control variables. To investigate the controlled associations, we used a structural equation model in which we regressed life satisfaction on physical activity, nutritional awareness, subjective stress and control variables. Parcels (test halves) were computed to represent the latent constructs. The model was based on maximum likelihood estimates and predictors were allowed to be correlated.

### Results

The descriptive statistics of all relevant variables and bivariate correlations are shown in Table 1. As expected, the amount of physical activity and nutritional awareness were positively correlated with life satisfaction. Subjective stress was negatively associated with life satisfaction, but positively with the consumption of less healthy food.

**Figure 1:**
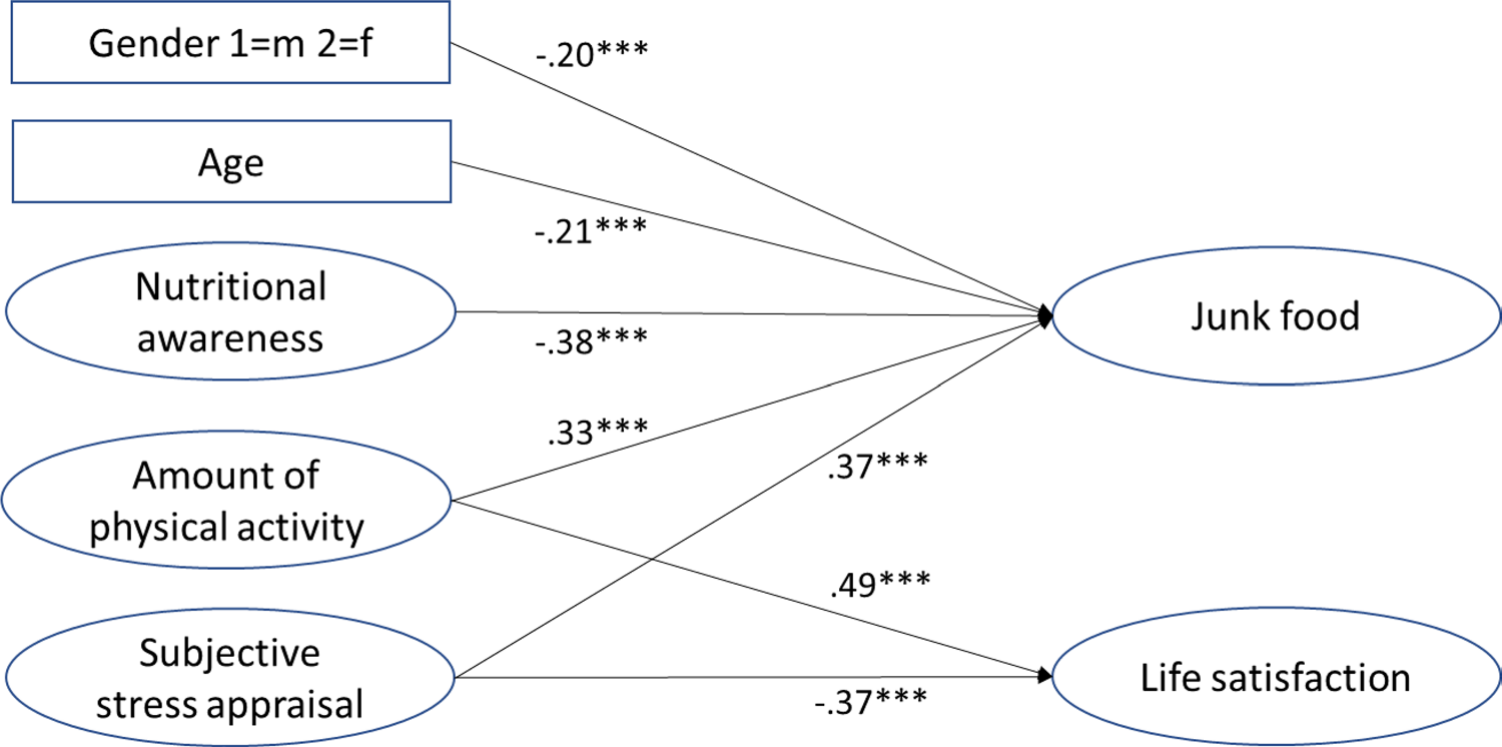
Structural equation model: associations with junk food and life satisfaction after controlling for age and gender. χ^2^(39) = 102.22; *p* < 0.01; RMSEA = 0.049; NNFI = 0.97; CFI = 0.98; AGFI = 0.95; ***p < 0.001. Depicted are standardized path coefficients.

Because physical activity and nutritional awareness were correlated, and the degree of food consumption was different depending on age and gender, we conducted a controlled analysis. In a structural equation model, we regressed life satisfaction and less healthy food consumption on age, gender, subjective stress, physical activity, and nutritional awareness to show the unique contributions of stress and health-related lifestyle variables after controlling for sociodemographic variables (see Fig. 1). Results showed that the association between physical activity and life satisfaction remained significant after controlling for other variables; the association between nutritional awareness and life satisfaction, however, was not significant. The expected association between subjective stress and the consumption of less healthy foods remained significant.

**Figure 2:**
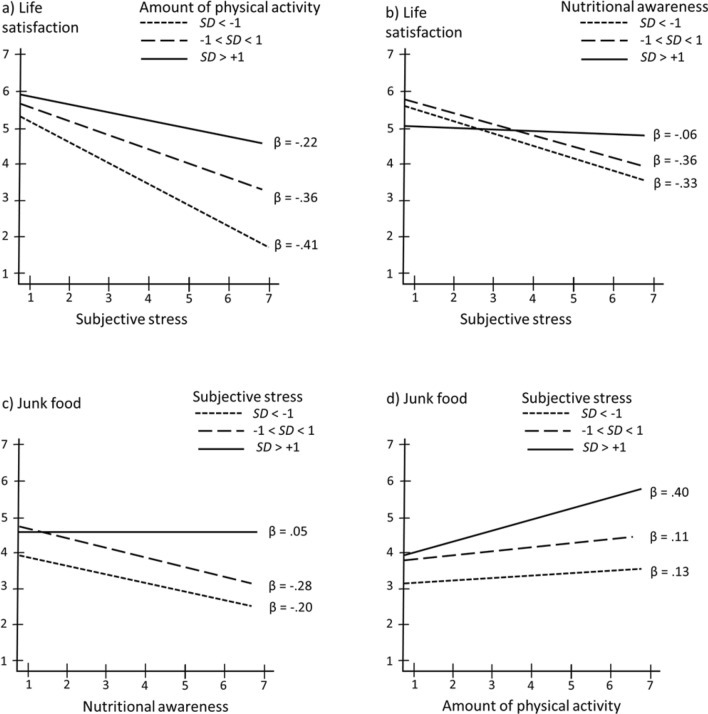
Moderating effects. Life satisfaction as a function of subjective stress and (**a**) physical activity, (**b**) nutritional awareness. Unhealthy nutrition as a function of subjective stress and (**c**) physical activity, (**d**) nutritional awareness.

In the third step, we tested the adaptive function of physical activity and nutritional awareness. Because we were interested in the moderating, stress buffering-effects of both variables, we used *z*-transformed scores to compute the interaction terms Subjective Stress x Physical Activity and Subjective Stress × Nutritional Awareness. Then we computed two moderated regression analyses (e.g., Subjective Stress, Physical Activity, Subjective Stress × Physical Activity → Life Satisfaction). As expected, the predictive value of subjective stress on life satisfaction was moderated by physical activity (β_Subjective Stress × Physical Activity_ = 0.10, *p* < 0.01) and nutritional awareness (β_Subjective Stress × Nutritional Awareness_ = 0.09, *p* = 0.015). Figure 2a,b depict the interaction effects. The negative associations between subjective stress and life satisfaction were less when physical activity and nutritional awareness were high.

To test our final hypothesis, we computed the interaction between subjective stress and nutritional awareness using a regression model Subjective Stress × Nutritional Awareness → Less Healthy Food. The interaction was significant (β _Subjective Stress × Nutritional Awareness_ = 0.07, *p* < 0.05). As expected, the negative association between nutritional awareness and less healthy food consumption was absent when the degree of subjective stress was high (see Fig. 2c).

Because the correlation between physical activity and less healthy food was, in contrast to our expectations, significant and positive, we also examined the interaction effect with subjective stress (Subjective Stress × Physical Activity → Less Healthy Food). The interaction effect was significant (β = 0.09; *p* < 0.014), showing that in times of less stress, the relationship between physical activity and junk food was low, however, in times of high subjective stress, the correlation was high (see Fig. 2d).

In sum, results of *Study 1* showed that the amounts of physical activity and nutritional awareness are correlated with life satisfaction; the association with nutritional awareness was small and failed to remain significant in a controlled analysis. Physical activity and nutritional awareness moderated the relationship between stress appraisals and general life satisfaction (stress-buffer effect). The relationship between nutritional awareness and less healthy food consumption was moderated by the degree of subjective stress. Interestingly enough, in contrast to the hypothesis, physical activity is correlated with less healthy food consumption. Because we did not differentiate between the situations in which people exercise (i.e. whether they do sport or have an active occupation), we conducted *Study 2* to differentiate this more precisely. We also wanted to test whether the interactions can be replicated.

## Study 2

### Methods

#### Participants

Data collection took place, again in Germany, from the second half of 2022 until March 2023 and was similar to that of *Study 1*. The recruiting process was the same as described for *Study 1*. Responses from seventeen of the 642 participants were not considered due to the questionable validity of the responses (unreasonably fast completion, incomplete or monotonous responding). The remaining participants (*N* = 625) ranged in age from 18 to 71 years with an average age of 43.09 years (*SD* = 15.98). More than half were female (52%), 47% were male, *n* = 4 diverse. There was no significant age difference between men and women, *t*(621) = 0.15, *p* = 0.88. Most participants were currently employed (78%), 22% were jobless, house-husbands/housewives, or retired. The body mass index was on average 26.16 (*SD* = 4.93). Fifty percent practise no sport regularly, 24% one, and 26% at least two.

#### Measurements

Subjective stress and life satisfaction were assessed as decribed in *Study 1*. Reliabilities were good (see Table 2). The measure of physical activity was modified in order to differentiate between occupational-related activities, and daily activities and sport.

**Table 2:** Descriptive statistics and bivariate correlations.

Variable	M (SD)	Range	α	Skewness/kurtosis	(2)	(3)	(4)	(5)	(6)	(7)	(8)	(9)	(10)
(1) Age	43.06 (15.99)	18–71	–	0.08/− 1.41	− 0.01	− 0.03	− 0.04	0.05	0.15***	0.20***	− 0.23***	− 0.22***	− 0.18***
(2) Gender 1 = m, 2 = f			–	− 0.01/− 1.65	1	0.04	− 0.10*	0.12**	− 0.03	0.07	− 0.09*	0.06	− 0.05
(3) Nutritional awareness	4.43 (1.06)	1–7	0.72	− 0.56/0.38		1	− 0.33***	0.41***	− 0.02	0.12**	0.32***	− 0.01	0.17***
(4) Less healthy nutrition	3.29 (1.12)	1–7	0.64	0.45/0.09			1	− 0.22***	0.07	0.13**	− 0.12**	0.23***	− 0.14***
(5) Healthy nutrition	5.21 (1.45)	1–7	0.73	− 0.55/− 0.47				1	0.05	0.13**	0.14***	− 0.13**	0.22***
(6) Activity at work^a^	3.06 (1.79)	1–7	0.68	0.54/ − 0.82					1	0.30**	0.01	0.06	− 0.02
(7) Leisure activity^b^	4.92 (2.71)	1–11.83	–	0.68/− 0.12						1	0.16***	0.08*	− 0.12**
(8) Sports^b^ activity	2.59 (1.95)	1–8.06	–	0.93/− 0.29							1	0.04	0.21***
(9) Subjective stress appraisal	3.53 (1.13)	1–7	0.93	0.09/ − 0.31								1	− 0.31***
(10) Life satisfaction	4.93 (1.35)	1–7	0.90	− 0.85/0.25									1

*N* = 628; ^a^*N* = 489; ^b^a square root transformation was conducted to improve the departure from normality.

##### Facets of physical activity

Three domains of the Physical Activity, Exercise, and Sport Questionnaire of Fuchs et al.^17^ were used to measure physical activity. *Daily activities*: Participants were asked how many days they had performed eight daily activities in the last four weeks, and how many minutes per day. We computed the sum score across activities. *Sport*: Participants were asked if they had regularly done sport in the past four weeks. For each of the sport mentioned, they were asked to indicate how many days and how many minutes per day on average. Occupational activities were assessed with two items: “Your occupational activities include (a) seated activities, (b) intensive activity” (none—a lot). We recoded the first item and computed the mean value (α = 0.68).

##### Nutritional awareness

Participants were asked to rate how well each item (“I follow a nutrition concept (e.g., vegan, vegetarian, low carb)”; “I pay attention to the nutrients and ingredients that are contained in foods”; “I watch what I eat”) applied to themselves in general on a seven-point Likert scale (*1* = not at all, *7* = exactly). Cronbach’s alpha was 0.77.

##### Less healthy foods/junk food

Participants were asked how often they had eaten the following foods in the past four weeks (*1* = very seldom to *7* = very often): (1) Fast food (e.g., burger, french fries), (2) Junk food/sweets, (3) Junk food/snacks, (4) Meat, and (5) Instant meals (α = 0.78). A surprising result of Study 1 was that activity was correlated with the consumption of less healthy food. To examine this in more detail, we used two indicators of *healthy food* (fruits, vegetables) and computed the mean value (α = 0.73).

As in *Study 1*, the descriptive statistics and bivariate correlations between the main variable were computed (see Table 2). Because daily activities and sport had positive skewness, a square root transformation was undertaken to improve the normality distribution after setting the smallest value to one^48^. To investigate the controlled associations, we used a structural equation model in which we regressed life satisfaction on facets of physical activity, nutritional awareness, subjective stress and control variables.

### Results

As expected, nutritional awareness was positively correlated with life satisfaction; the correlation was small. In terms of the amount of physical activity, the results showed that sport correlated with life satisfaction. The association between leisure activity and life satisfaction was small, but negative (*r* = − 0.11, *p* < 0.001); work activity was not associated with life satisfaction. Subjective stress during the last four weeks was negatively associated with life satisfaction, but positively with the consumption of less healthy food. The results showed that less healthy food consumption was differentially associated with facets of activity; the correlation with leisure activity was positive, the correlation with sport negative.

**Figure 3:**
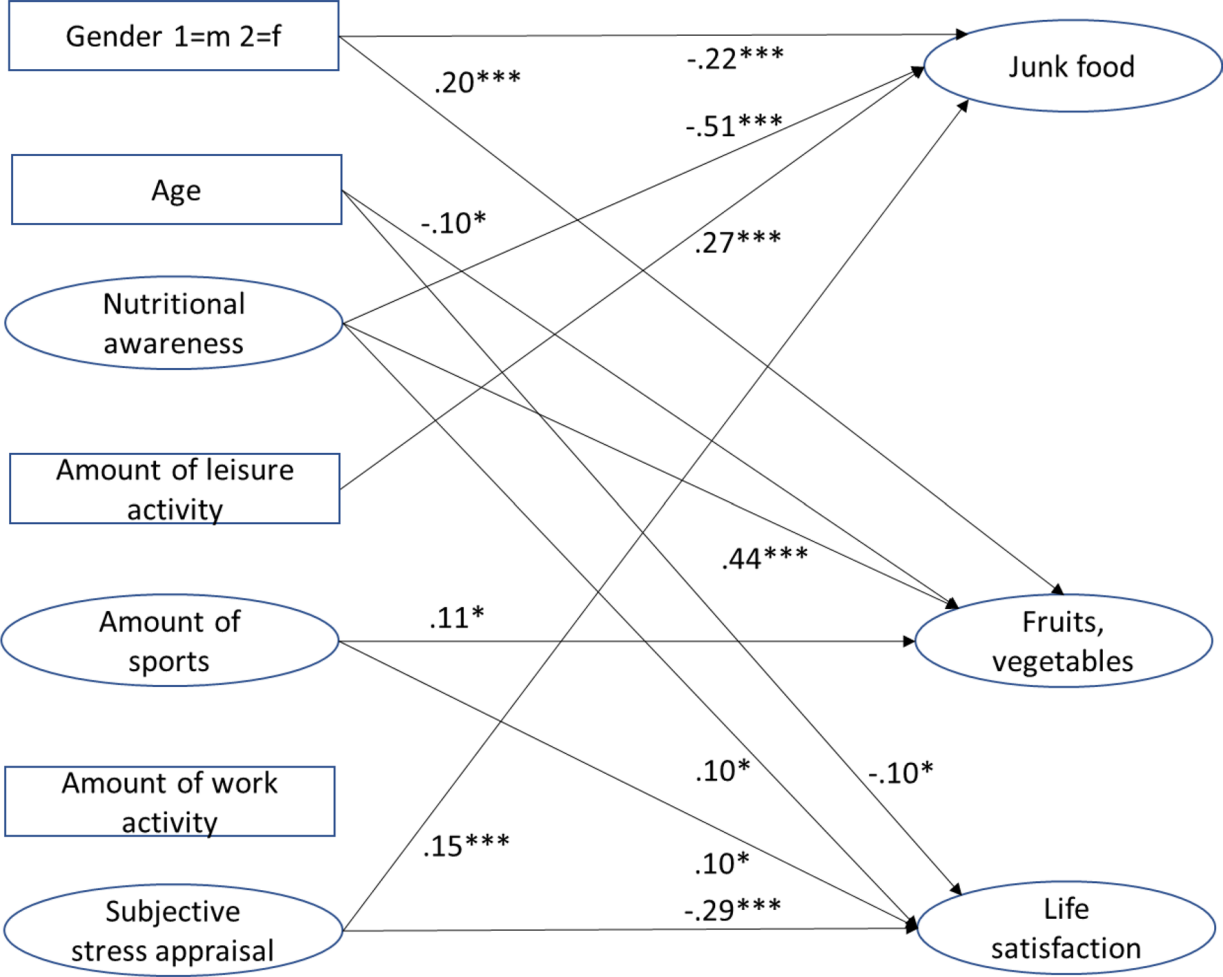
Structural equation model: associations with junk food, fruits/vegetables, and life satisfaction after controlling for age and gender. χ^2^ (66) = 156.62; p < 0.001; RMSEA = 0.056; NNFI = 0.94; CFI = 0.96; AGFI = 0.92; *p < 0.05; **p < 0.01; ***p < 0.001. Depicted are standardized path coefficients.

In the next step, we tested the associations between subjective stress, facets of physical activity, and nutritional awareness on food consumption and life satisfaction, after controlling for age and gender. This structural equation model was computed with those 489 participants who were currently working. Life satisfaction, healthy, and less healthy food consumption were regressed on the main variables, age, and gender (see Fig. 3). The main predictor of life satisfaction was subjective stress; the predictive value of nutritional awareness remained significant, but was small. The predictive value of the single facets of physical activity was reduced, the strongest was the unique association between work activity and life satisfaction. In terms of physical activity dimensions, the results showed that consumption of less healthy food was associated with the amount of leisure activity, but not with that of sport or work activity. The association between consumption of fruits or vegetables and leisure activity was not significant.

Next, we tested the moderation effects and computed the interaction terms between subjective stress and nutritional awareness or facets of physical activity. As expected, and similar to *Study 1*, the correlation between subjective stress and life satisfaction was moderated by nutritional awareness (β_Subjective Stress × Nutritional Awareness_ = 0.09, *p* = 0.01). The buffering effect was also significant for leisure activity (β _Subjective Stress x Leisure Activity_ = 0.15, *p* < 0.001), for work activity (β_Subjective Stress × Work Activity_ = 0.10. *p* < 0.05), but not for sport (β _Subjective Stress × Sport_ = − 0.01, *p* = 0.95). The effects were similar to those of *Study 1*; the associations between subjective stress and life satisfaction were lower when nutritional awareness, and the degree of activity in leisure time were high.

To test our final hypothesis, we computed the regression model Subjective Stress x Nutritional Awareness → Less Healthy Food. The interaction was marginally significant (β_Subjective Stress × Nutritional Awareness_ = 0.07, *p* < 0.06). As expected, the negative association between nutritional awareness and less healthy food consumption was absent when the degree of subjective stress was high (see Fig. 2c).

Finally, we examined the surprising finding that in times of high stress physical activity is more strongly associated with less healthy food consumption (Stress × Physical Activity → Less Healthy Food). The interaction was significant (β_Subjective Stress × Daily Activity_ = 0.11, *p* < 0.01) and a post-hoc analysis showed that the association between less healthy food consumption and daily activity was high in times of high stress. The protective function of sport decreased and the negative correlation dropped close to zero when the degree of subjective stress was high (β_Subjective Stress × Sport_ = 0.08, *p* = 0.03). We found no interaction with work activity.

## Discussion

Although there is ample evidence that physical activity and nutrition are associated with well-being, their protective function in times of stress is less frequently examined. The present studies focused on both as sample cases for resilience. We investigated the phenomenon in terms of risk profiles and life satisfaction. As expected, both studies showed that physical activity buffered the negative relationship between current subjective stress and general life satisfaction. *Study 2* provided evidence that in particular leisure activity and work activity exhibited an adaptive value. Altogether, it was shown that both facets of a healthy lifestyle served as stress-moderating resources, which would be expected from relational models of resilience (e.g.,^9^). The interpretation of correlative data must remain speculative, but it could be that temporary stress experiences do not extend to the global evaluation of life, when people are mindful and pursue health-related goals.

A greater amount of sport, which possibly requires a higher degree of intention and commitment than other activities, did not serve as a stress buffer. The motivation for doing athletic activities could be revealing here. Some persons who do many athletic activities may have high ambitions or demands on their own performance, which could limit the protective effects of the activities on life satisfaction. The missing interaction is in line with the results of Meyer et al.^23^, who found a stress-buffering effect of sport and exercises only when intrinsic motivation was high.

However, it was sport that showed the strongest prediction of life satisfaction in *Study 2*. The association between physical activity and life satisfaction was different in the two studies. *Study 1* provided a clear correlation, which is comparable to the results of other studies (e.g.,^11^). *Study 2* demonstrated no evidence for a strong association between physical activity and mental well-being (see also^10,18,23^). Here, too, the reasons why people exercise could be informative: whether it is because of a positive attitude towards health or whether occupational circumstances require more activity.

Besides physical activity, nutritional mindfulness also moderated the negative association between subjective stress and life satisfaction; bivariate correlations between nutritional awareness and life satisfaction were weak. Together, the results allow the interpretation that a protective function of exercise and nutritional awareness lies in its stress-buffering effect, in that healthier lifestyles, and being mindful of them, protect against overall loss of well-being. The evidence is less clear for main effects (i.e. direct relations to life satisfaction).

Another question arises from the present study related to the consumption of unhealthy food during times of increased stress and the assumption that dietary intentions are less effective then. In both studies, the level of stress experienced correlated with the consumption of less healthy food. The pattern of findings is consistent with many studies (e.g.,^49,50^) that reflect the negative side of stress eating. The interaction analyses also illustrated a nutritional behavior mechanism that draws attention to the difficulty of dissolving the negative relationship between nutritional awareness and unhealthy diets during stressful times. Positive nutritional intentions are no longer implemented adequately.

The somewhat surprising finding from *Study 1* that a lot of physical activity is associated with a less healthy diet (even more pronounced in the case of stress) could be analysed in more detail in *Study 2*. Apparently, only leisure activity is associated with unhealthy eating. In contrast, we found a protective pattern for sport: Particularly in times of less stress, sport was negatively associated with the consumption of less healthy food. With a lot of stress, however, the protective pattern decreased. Possible reasons are that people may have less time or willpower to implement their positive health-related resolutions.

In summary, the results showed that health-related lifestyles (sport, nutritional awareness) are not (only) directly related to the experienced stress, but are also implemented inefficiently in times of high stress.

## Limitations and future research

We took a stress-buffering approach as a sample case of resilience and concentrated on general life satisfaction as the indicator of positive adaptation in the face of stress. Other variables (e.g., subjective health as an outcome) or adverse situations (critical life events) could also be possible means to examine a resilience constellation. Both studies are based on cross-sectional, self-report data and thus any possible mutual influences could be plausible. One example might be differences in the regions where people live (e.g., rural or urban areas), which might affect not only activity levels but also other aspects of daily life. Another issue that might have arisen in giving exact answers to questions about eating habits was that the food items might not have reflected the actual food consumption or may have had possible confounding factors (e.g., meat in fast food). In future studies, the temporal sequences and causal relations should be examined. It would be important to examine whether an increase in nutritional and physically-related behavior would predict an increase in resilience and a decrease in less healthy nutrition. Intervention studies can provide evidence as to whether both physical and nutritional activities complement one another and lead to an accumulated resilience effect.

## Conclusions

An ambivalent association between healthy lifestyle and life satisfaction became evident. Both studies showed that physical activity and nutritional awareness buffer the negative association between stress and life satisfaction. This is a sample case of resilience: the resource is for individuals to pursue a life content and a health-related lifestyle in which resilience is expressed in such a way that current stress does not reduce general life satisfaction. In everyday life, however, it is important to notice the multifaceted function of a healthy lifestyle, also its vulnerability. For health counselling, consideration should be given to how a healthy lifestyle can be integrated into everyday life. One possible interpretation of the cross-sectional results is that a concept of a healthy lifestyle provides motivation for people not to slacken under stress but to rather continue to pay attention to their health; and this contributes to life satisfaction. Important tasks of health counselling would be to identify dangers of malnutrition and to point out eating that counteracts diets. Both studies showed not only that stress is a risk situation associated with unhealthy eating habits, but also that in times of stress, exercise and nutrition do correlate negatively, however, to a lesser degree with junk food consumption. Nutritional counselling should keep in mind that a risk lies in the intention-behavior gap (in other words—one has good intentions, but still eats unwisely). In times of enhanced stress, less healthy food consumption may satisfy in the short term and might be seen as a kind of savouring of life, but endangers the resilience of the body in the long term.

### Ethics approval and consent to participate

The study was conducted according to the guidelines of the Declaration of Helsinki and approved by the Ethics Committee of the University of the Bundeswehr Munich, Germany (EK UniBw M 23-06, 12/16/2022). All participants provided informed written consent prior the survey.

## Data Availability

Data is provided within the manuscript. The datasets used and analyzed during the current study are available from the corresponding author on reasonable request.
